# Increased chronic disease prevalence among the younger generation: Findings from a population-based data linkage study to inform chronic disease ascertainment among reproductive-aged Australian women

**DOI:** 10.1371/journal.pone.0254668

**Published:** 2021-08-18

**Authors:** Melissa L. Harris, Nicholas Egan, Peta M. Forder, Deborah Loxton

**Affiliations:** Centre for Women’s Health Research, University of Newcastle, Newcastle, Australia; Osakidetza Basque Health Service, SPAIN

## Abstract

**Background:**

Chronic disease represents an ongoing public health challenge in Australia with women disproportionately affected and at younger ages compared to men. Accurate prevalence and ascertainment of chronic disease among women of reproductive age at the population level is essential for meeting the family planning and reproductive health challenges that chronic diseases pose. This study estimated the prevalence of chronic disease among younger Australian women of reproductive age, in order to ascertain key conditions that would benefit from targeted family planning support strategies.

**Methods and findings:**

Population-level survey data from the 1973–78 and 1989–95 cohorts of the Australian Longitudinal Study on Women’s Health were linked to health service use, pharmaceutical, cancer and cause of death data to ascertain the prevalence and chronic disease trends for ten chronic health conditions associated with poor maternal and foetal outcomes. Individual chronic disease algorithms were developed for each chronic disease of interest using the available linked datasets. Lifetime prevalence of chronic disease varied substantially based on each individual data source for each of the conditions of interest. When all data sources were considered, all conditions with the exception of mental health conditions were higher among women in the 1973–78 cohort. However, when focused on point prevalence at similar ages (approximately 25–30 years), the chronic disease trend for women in the 1989–95 cohort was substantially higher, particularly for mental health conditions (70.4% vs 23.6%), diabetes (4.5% vs 1.3%) and multimorbidity (17.9% vs 9.1%).

**Conclusions:**

Given the low concordance between individual data sources, the use of multiple data sources are recommended for chronic disease research focused on women of reproductive age. In order to reduce the increasing chronic disease and multimorbidity trend among women, strategic chronic disease interventions are required to be implemented in childhood and adolescence to ensure the long-term health of not only current but also future generations.

## Introduction

Chronic disease represents an ongoing public health challenge. It contributes substantially to global healthcare expenditure and disease burden in terms of health service use, hospitalisations as well as costs associated with treatment regimens [1, 2]. Australia, in particular, is facing a chronic disease crisis. In 2011, chronic disease was reported as an underlying cause in 90% of deaths [3]. Despite the increased focus on preventative health strategies, chronic disease rates continue to rise with estimates from the 2017–18 National Health Survey (which provides a snapshot of Australia’s health) suggesting that half of adults have at least one chronic health condition (up from one-third in 2007–08) [1, 4]. Women in particular appear to be disproportionately affected, and at younger ages compared to men [5]. Of the common conditions listed in the recent National Health Survey, 26% of women reported one chronic disease and 23% reported two or more conditions (multimorbidity). Importantly, while 83% of Australian women reported chronic disease when aged over 65 years, 43% reported chronic disease during their reproductive years (i.e., <45 years). Findings from population-based cohort studies support the increasing prevalence of chronic diseases such as diabetes across successive generations [6].

Accurate prevalence and ascertainment of chronic disease among women of reproductive age at the population level is essential for not only public health surveillance, policy and healthcare planning but also meeting the family planning and reproductive health challenges that chronic diseases pose [7]. While a number of data sources have been used for monitoring chronic disease trends such as population-based surveys, medical chart review, disease registries and administrative healthcare databases (e.g., primary health care, hospitalisation and pharmaceutical), there is no clear reference standard for the ascertainment of chronic disease.

Epidemiological research focused on ascertainment techniques among older populations have demonstrated that a single data source is insufficient to accurately capture chronic diseases. All data sources have limitations (with variable agreement between sources depending on the disease of interest), which may contribute to unreliable case ascertainment and biased prevalence and incidence estimates [8–14]. Even in diseases such as diabetes, which can be clearly identified in administrative data, disease prevalence and incidence may still be under-reported. For example, in the case of type 2 diabetes, one-third of cases are controlled by diet and lifestyle alone leading to fewer diabetes specific records in administrative data [15]. Therefore, in order to increase the validity of chronic disease ascertainment, use of multiple data points through linkage of different data sources (including the development of tailored algorithms on a disease specific basis) is increasing [11, 16–22].

In Australia, chronic disease is managed through a tax-funded universal healthcare system, which provides researchers access to rich and long-term health service and pharmaceutical claims data. Additionally, the Australian Government has funded the Australian Longitudinal Study on Women’s Health (ALSWH) since 1996, to follow a cohort of women born 1973–78 through their reproductive years, over two decades, collecting information on their health outcomes and use of health services, as well as social, behavioural and economic factors which influence health. This national longitudinal study also recruited a new cohort in 2012, aiming to collect similar information on the next generation of younger Australian women [23, 24]. As these data are routinely linked to administrative health records, there is an ideal opportunity to estimate the prevalence of chronic diseases among women of reproductive age using multiple data sources, and to assess the concordance between available data sources. This approach has not previously been undertaken specifically for women of reproductive age. With chronic disease an ongoing public health issue and with chronic disease development occurring earlier in life, particularly for women, it has implications for the delivery of family planning support strategies and preconception contraceptive counselling as well as policy implications for advancing reproductive health. The aim of this paper is to estimate the prevalence of chronic disease among younger Australian women of reproductive age, in order to identify conditions that would benefit from targeted family planning support strategies and preconception contraceptive counselling interventions.

## Materials and methods

### Data sources

#### ALSWH survey

**Fig 1:**
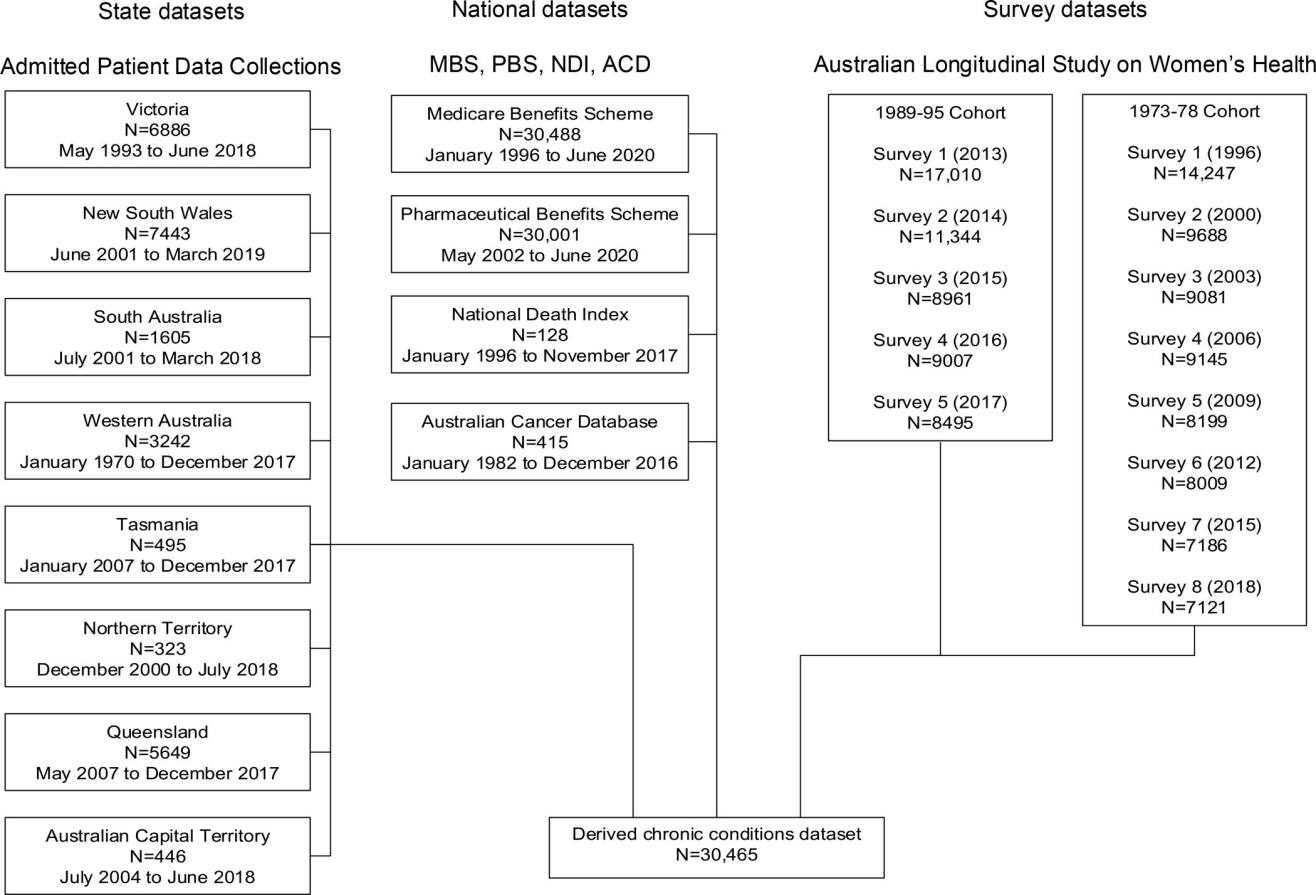
Relationships between data sources for case ascertainment of 10 key chronic conditions.

Chronic disease cases among reproductive-aged women were ascertained using data collected from women in the 1973–78 and 1989–95 cohorts of the ALSWH, a national population-based study of health and wellbeing among Australian women. Women from the 1973–78 cohort were randomly sampled through the Medicare Australia database in 1996 when aged 18–23 years (N = 14,247), with women from rural and remote areas sampled at twice the rate as those from urban areas [23, 25]. These women have been surveyed on a roughly three-year rolling schedule with follow-up surveys administered in 2000 (Survey 2), 2003 (Survey 3), 2006 (Survey 4), 2009 (Survey 5), 2012 (Survey 6), 2015 (Survey 7) and 2018 (Survey 8). ALSWH recruited a new cohort of eligible women aged 18–23 years in 2012–2013 (N = 17,010) who completed annual online health surveys until 2017, and a sixth survey in 2019. To meet the needs of this generation, the newest ALSWH cohort was recruited through an open recruitment strategy involving a mix of online and offline methods [24]. Both cohorts of women have been found to be largely representative of women in their age group, with a slight over-representation of Australian-born and tertiary educated women [25, 26]. For the majority of women in these cohorts, survey data are available for linkage to a number of health administrative datasets. For the purposes of this study, ALSWH survey data were linked to the following health administrative databases: state and territory-based Admitted Patient Data Collections, National Death Index, Pharmaceutical Benefits Scheme, Medicare Benefits Schedule, and the Australian Cancer Database. The relationships between these data sources are presented in Fig 1.

#### Admitted Patient Data Collections (APDC)

Data on hospitalisations were obtained from individual state and territory-based datasets which provide information on all public and private admitted patient services provided for each resident, with the exception of the Australian Capital Territory [ACT], Northern Territory [NT] South Australia [SA] and Tasmanian [TAS] collections which only cover nominated public hospitals. These databases contain information on, but are not limited to, admission and separation dates, procedures undertaken as well as diagnoses related to the admission. Diagnoses are coded according to the Australian Modification of the International Statistical Classification of Diseases and Related Problems (ICD-AM) for a primary diagnosis and up to an additional 54 secondary diagnoses (depending on the state and coding version). APDC records were linked to ALSWH participants via a master linkage key using probabilistic linkage methods for the following states/territories and time periods: a) June 2001 to March 2019 for New South Wales (NSW); b) July 2004 to June 2018 for the ACT; c) May 2007 to December 2017 for Queensland (QLD); d) May 1993 to June 2018 for Victoria (VIC); e) July 2001 to March 2018 in South Australia (SA); f) January 1970 to December 2017 in Western Australia (WA); g) January 2007 to December 2017 in TAS; and h) December 2000 to July 2018 in the NT.

#### National Death Index (NDI)

Causes of death and date of death were determined from the National Death and Morbidity Indexes based on probabilistic matching of person level information such as name, address, gender, state, and date of birth for the period January 1996 to November 2017 [27].

#### Pharmaceutical Benefits Scheme (PBS)

The PBS database was used to identify ongoing prescription medications for chronic disease management related to each of the conditions of interest. The PBS provides Commonwealth subsidised prescriptions medications for eligible Australian citizens and residents as well as foreign visitors covered by a Reciprocal Health Care agreement. Prior to April 2012 only prescriptions which carried a government subsidy and were above co-payment were recorded. From April 2012, all above and below co-payment government subsidised medications were captured through the PBS database. PBS data for ALSWH participants were deterministically linked using the individuals’ unique Medicare number [28]. PBS data were included for the period May 2002 to June 2020.

#### Medical Benefits Schedule (MBS)

The MBS database provides information regarding claims for medical services by Australian citizens, permanent residents and foreign visitors covered by a Reciprocal Health Care agreement as part of Australia’s tax-funded universal health care system. The MBS database provides comprehensive information regarding a range of services, diagnostic procedures and tests provided by general practitioners, psychologists, allied health or medical specialists. MBS data were linked in a similar manner to PBS data for the period January 1996 to June 2020.

#### Australian Cancer Database (ACD)

The ACD is a national compilation of data from all state and territory-based cancer registries which are routinely collected under legal mandate. As a result, this database provides near complete national information on malignant neoplasms (i.e., excluding non-melanotic skin cancer) recorded from public and private hospitals (including inpatient admissions and day stay procedures), radiation oncology units, residential aged care, pathology laboratories, and outpatient units. The ACD contains pertinent information regarding cancer type, date of diagnosis, age at diagnosis, topography and morphology, tumour size as well as information surrounding survival [29]. Cancer information for ALSWH participants were probabilistically linked for the period January 1982 to December 2016 for all states and territories (except for NT, which is only linked to 2015).

### Chronic disease ascertainment

Chronic diseases were identified following consultation with the literature on adverse maternal and foetal outcomes, Australia’s national health priority areas, the UK medical eligibility for contraception and the ability to ascertain the cases in the available datasets [30–32]. As the premise of the study was to ascertain key chronic health conditions that would benefit from preconception contraceptive counselling interventions, chronic diseases such as polycystic ovary syndrome and endometriosis were excluded from the chronic disease definition due to their impact on fertility [33, 34]. As such, the final set of conditions to be ascertained included: i) diabetes; ii) cardiac disease; iii) chronic kidney disease; iv) asthma; v) autoinflammatory arthropathies and connective tissue disease; vi) inflammatory bowel disease; vii) multiple sclerosis; viii) thyroid disease; ix) mental health conditions; and x) cancer. Where possible, both congenital and acquired forms of the disease were included. The definition for cardiac disease was based on the Faculty of Sexual and Reproductive Healthcare Clinical Guidance Report and included conditions such as conduction disorders, dysrhythmias, congestive heart failure, cardiomyopathy, ischaemic heart disease, rheumatic heart disease, heart defects and pulmonary circulation disorders. Vascular diseases such as cerebrovascular disease, peripheral vascular disease and venous thromboembolism were considered in the context of cardiac complications only and as such were excluded [35]. Hypertension, a pre-cursor to acquired heart disease, is increasing among people under the age of 50 years largely due to increasing rates of obesity. Hypertension (uncomplicated and complicated forms) was included with cardiac disease due to its impact on maternal and foetal outcomes [36]. Mental health conditions were focused on the following disorders: mood (e.g., major depressive disorder, bipolar disorder), anxiety and stress (e.g., panic disorder, obsessive compulsive disorder), psychotic (e.g., schizophrenia), personality (e.g., borderline personality disorder) and eating (e.g., anorexia nervosa). All malignant forms of cancer were included (i.e., excluding in situ cancers of the breast and skin).

#### Algorithm development

Individual algorithms were developed for each chronic disease of interest, using the available linked datasets. ICD-AM codes supplied for primary and secondary diagnoses in the inpatient hospital datasets were used to identify chronic conditions of interest. Due to the time frame of the data coverage from the linked data sources, both ICD-10AM and ICD-9-AM codes were used for case ascertainment, as appropriate for each data source. For each chronic condition, ICD codes from the Charlson and Elixhauser indices [37–39] were used for case ascertainment with further refinement after consultation with clinical experts for each condition (see S1 Table). Deaths related to the conditions of interest were identified in a similar fashion to those used for inpatient hospital records (APDC). For PBS data, case identification was performed using the World Health Organization’s Anatomical and Therapeutic Chemical Classification (ATC) codes [40]. Medications which are typically indicated in the treatment of the selected conditions were identified using the Australian Medicines Handbook, with confirmation from clinical disease experts [41].

Using the ALSWH survey data, case ascertainment of chronic diseases was based on participants’ self-report of diagnosis or treatment of the conditions of interest. The time frame in which the women were asked to report this information varied by survey and cohort, with conditions containing sub-types (e.g., diabetes and mental health conditions) being asked with increasing precision over time (see S2 Table). While direct questions about more prevalent chronic conditions were included in questionnaires over time (e.g., diabetes, heart attack), information concerning other conditions of interest may have been provided by the participant themselves in the free-text comments area when asked to specify any other major illness not listed. As a result, the free-text comments were also used for case ascertainment, using a search on keywords (including colloquial and misspelled variants) based on the ICD codes for each of the disease groups (e.g. diab*, daib*, insulin, kidney, crohns, etc).

In general, for inclusion as a chronic disease ‘case’, all conditions with the exception of cancer required either: (a) one indication in either the APDC, MBS or NDI; or (b) self-report in two ALSWH surveys; or (c) two or more medications prescribed within a 12-month period, reported in two separate calendar years (as it was assumed that a chronic condition would require regular ongoing medications). Where a medication had multiple indications for treatment (e.g., metformin [A10BA02] is primarily prescribed in the treatment of diabetes but can also be prescribed in the treatment of PCOS), the medication was included if there was an indication for that condition from another data source (e.g., was also captured through MBS data or via survey self-report). For conditions which include transient gestational/antenatal forms, pregnancy-specific ICD 10 codes and medications prescribed during times of pregnancy (based on each child’s date of birth) were excluded from the chronic disease definition. For medication use, this included a 40-week exclusion period for diabetes and depression/anxiety and 20-weeks for gestational hypertension [42]. Where MBS data were available, one item related to the management of the conditions (and not purely diagnostic) was required for inclusion (with the exception of diabetes). An example algorithm for diabetes is shown in Table 1, with case ascertainment algorithms for the remaining conditions using multiple data sources provided in S3 Table.

**Table 1:** Case ascertainment algorithm for diabetes.

	ELIGIBILITY CRITERIA
**ALSWH SURVEYS**	Diabetes self-reported in at least two surveys
**MBS**	Reported once or more for diabetes annual cycle; eye exam, specific for patients with established diabetes; and allied health group service, specific for patients with established diabetes
	**OR**
	*2 or more HbA1c tests related to management of established diabetes within a 12- month period
**PBS** ^ç^	Reported twice or more within a 12-month period in two calendar years
**COD**	Reported once
**APDC**	Reported once or more

Notes: ALSWH = Australian Longitudinal Study on Women’s Health; MBS = Medicare Benefits Schedule; PBS = Pharmaceuticals Benefits Scheme; COD = Cause of Death (from the National Death Index); APDC = Admitted Patient Data Collection.

*The MBS Service Incentive Program for diabetes care requires at least one HbA1c measurement per year. It is recommended that HbA1c testing is done every 6 months if meeting targets, or every 3 months if targets are not being met or if therapy has changed. Therefore, at least 2 HbA1c tests within a 12-month period was required for inclusion.

^ç^PBS: Excluding times of pregnancy (defined as 40 weeks), based on child’s dates of birth. This was to exclude women who may have been diagnosed & treated for gestational diabetes.

### Ethics

All data for this project were obtained from the ALSWH (see www.alswh.org.au/ for further details), approved under their Expression of Interest process (EOI A696) and provided in de-identified form. This project has ongoing ethical clearance from the University of Newcastle and University of Queensland’s Human Research Ethics Committees. Ethical approval for linkage of ALSWH survey data to the Admitted Patients Data Collections (APDC) was received from the NSW Population and Health Services Research Ethics Committee and other equivalent state and territory-based committees. Linkages to the National Death Index (NDI) and Australian Cancer Database (ACD) were approved by the Australian Institute of Health and Welfare Ethics Committee. Women provided explicit written consent to participate in the ALSWH as well as for linkage to de-identified administrative health records prior to 2005. From 2005, an ‘opt-out’ consent process was approved by the data custodians and relevant ethics committees for data linkage, with participants regularly reminded of this process. ALSWH participants who decline health record linkage are excluded from data linkage requests.

### Statistical analysis

The number of women with each condition of interest was ascertained using multiple data sources and the applicable algorithm. Concordance between data sources was calculated as the percentage agreement between each pair of sources among women with the specified condition. For example, if 100 women overall were determined to have diabetes, and 75 of those women had indications from both MBS and PBS, then the percentage agreement between MBS and PBS for diabetes was 75%.

Given that the case ascertainment algorithms provided a conservative measure of disease ascertainment using surveys (i.e., a minimum of two separate self-reports were required across surveys), a sensitivity analysis was conducted for each condition to examine the potential impact on disease prevalence from survey loss-to-follow-up. In modified algorithms for the sensitivity analyses, women were included as having a self-reported indication for the condition if the condition was reported at a single survey only which was also the woman’s last completed survey (i.e., they did not have a later opportunity to self-report via survey). Differences between the standard algorithm and the modified algorithm for each condition were compared. All data preparation and analyses were conducted in Stata 15.1 (College Station, TX: StataCorp LLC).

## Results

### Sample characteristics

**Fig 2:**
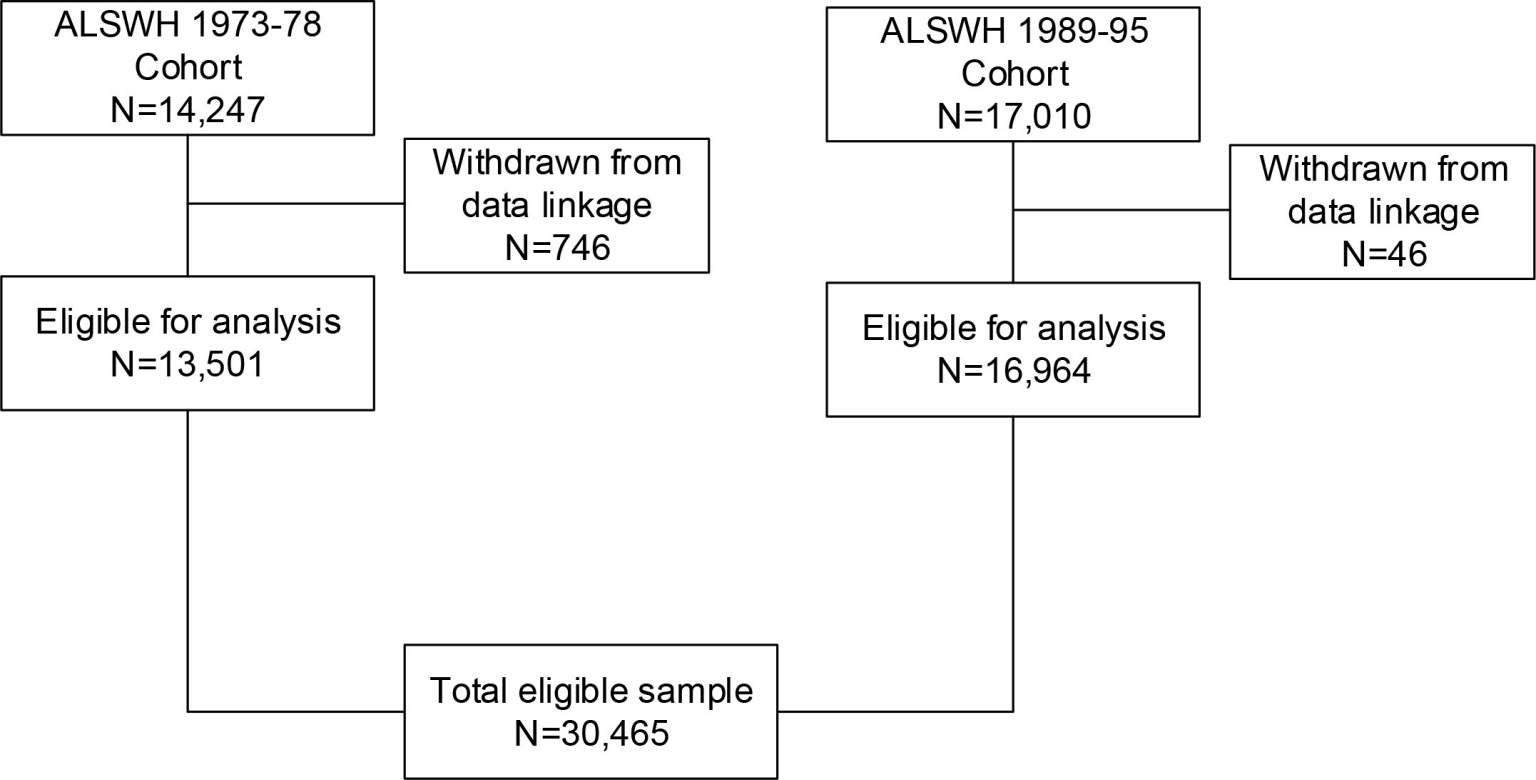
Flow diagram of eligible participants.

The 1973–78 cohort who completed the baseline survey in 1996 contained 14,247 women, of whom 13,501 (94.8%) did not opt out of data linkage and were eligible for inclusion in this analysis (see Fig 2). Similarly, only a small percentage (0.3%) of the 17,010 women from the 1989–95 cohort did not provide consent to data linkage, leaving 16,964 women eligible for analysis.

Baseline characteristics for women included in the analysis are shown in S4 Table and were obtained from the first survey completed, either in 1996 (1973–78 cohort) or 2012/2013 (1989–95 cohort). Most women in both cohorts were Australian born, unpartnered and lived in a major city at their baseline survey. A higher proportion of women from the 1989–95 cohort were overweight or obese (32.5% vs 21.9%), had achieved tertiary qualifications (21.9% vs 11.1%), were non-smokers (81.2% vs 52.1%) and rated their health as fair or poor (16.9% vs 12.2%) compared to the 1973–78 cohort.

### Concordance between data sources

The number and types of data sources used to ascertain cases varied across chronic diseases (Table 2). Between 28% and 70% of chronic diseases were ascertained through one data source only, depending on the condition. Diabetes had the highest prevalence of ascertainment through a single source (70%) while mental health conditions had the lowest prevalence (28%). Diabetes, autoinflammatory arthropathies and connective tissue disease, inflammatory bowel disease, and chronic kidney disease were the most likely to be ascertained through administrative data only, with 68% to 79% of cases identified in this way. However, the prevalence of each condition within each administrative data source differed depending on the condition. Diabetes was most frequently ascertained through the MBS, autoinflammatory arthropathies and connective tissue disease through the PBS and inflammatory bowel disease through the APDC. Multiple sclerosis had the highest proportion of cases identified through survey only, while mental health conditions, inflammatory bowel disease, and cancer had the highest proportion of cases identified through three or more data sources.

**Table 2:** Ascertainment of ten chronic disease conditions by June 2020 among younger Australian women across their reproductive years^a^, according to the number and types of data sources.

	Diabetes	Cardiac disease	CKD	Asthma	Arthrop	IBD	Thyroid disease	MS	Mental health	Cancer
	N	N	N	N	N	N	N	N	N	N
Total number of cases identified from any source	1670	1805	56	4974	866	236	840	260	19712	809
	**N (%)**	**N (%)**	**N (%)**	**N (%)**	**N (%)**	**N (%)**	**N (%)**	**N (%)**	**N (%)**	**N (%)**
Number of sources										
1	1171 (70)	1030 (57)	37 (66)	2271 (46)	645 (75)	98 (42)	576 (69)	195 (75)	5516 (28)	428 (53)
2	200 (12)	614 (34)	13 (23)	2073 (42)	192 (22)	78 (33)	236 (28)	46 (18)	7455 (38)	204 (25)
≥3	299 (18)	161 (9)	6 (11)	630 (13)	29 (3)	60 (25)	28 (3)	19 (7)	6741 (34)	177 (22)
Type of data source										
Survey	413 (25)	985 (55)	12 (21)	4064 (82)	267 (31)	76 (32)	226 (27)	217 (83)	13294 (67)	461 (57)
MBS	1448 (87)	150 (8)	16 (29)	644 (13)	NA	21 (9)	NA	NA	18424 (93)	NA
PBS	368 (22)	906 (50)	10 (18)	2976 (60)	770 (89)	156 (66)	731 (87)	72 (28)	7964 (40)	NA
APDC	410 (25)	712 (39)	42 (75)	692 (14)	78 (9)	188 (80)	175 (21)	55 (21)	2369 (12)	464 (57)
NDI*	1 (0)	14 (1)	2 (4)	4 (0)	1 (0)	0 (0)	0 (0)	0 (0)	9 (0)	37 (5)
ACD	NA	NA	NA	NA	NA	NA	NA	NA	NA	412 (51)
Women with survey only	44 (3)	313 (17)	4 (7)	1468 (30)	61 (7)	1 (0)	25 (3)	171 (66)	677 (3)	186 (23)
Women with admin. only	1257 (75)	820 (45)	44 (79)	910 (18)	599 (69)	160 (68)	614 (73)	43 (17)	6418 (33)	348 (43)
Women with survey and admin.	369 (22)	672 (37)	8 (14)	2596 (52)	206 (24)	75 (32)	201 (24)	46 (18)	12617 (64)	275 (34)

^a^ Eligible women from the Australian Longitudinal Study on Women’s Health (born 1973–78 or 1989–95).

CKD = Chronic Kidney Disease; Arthrop = Autoinflammatory arthropathies and connective tissue disease; IBD = Inflammatory bowel disease; MS = Multiple Sclerosis.

MBS = Medicare Benefits Schedule; PBS = Pharmaceuticals Benefits Scheme; APDC = Admitted Patient Data Collection; NDI = National Death Index; ACD = Australian Cancer Database.

* Across all conditions, only 68 indications were obtained from NDI, as there were only 128 deaths across the two cohorts during the observation period.

Low concordance between data sources was reported for all examined conditions (see Table 3), regardless of the sources examined. Survey and PBS had the highest concordance for several conditions, although the degree of concordance varied widely (from 5% for chronic kidney disease up to 46% for asthma). Overall, the highest concordance was found between the APDC and PBS for inflammatory bowel disease (49%).

**Table 3:** Concordance between data sources for case ascertainment of ten chronic disease conditions by June 2020 among younger Australian women across their reproductive years^a^.

Data sources	Diabetes (%)	Cardiac disease (%)	CKD (%)	Asthma (%)	Arthrop (%)	IBD	Thyroid disease (%)	MS (%)	Mental health (%)	Cancer (%)
						N (%)				
Survey and APDC	14	14	11	11	5	27	5	10	10	24
Survey and PBS	16	28	5	46	22	28	23	15	32	NA
Survey and MBS	18	4	4	10	-	3	NA	NA	62	NA
APDC and PBS	14	11	13	7	5	49	11	15	8	NA
APDC and MBS	17	4	14	2	NA	6	NA	NA	11	NA
MBS and PBS	17	2	7	8	NA	5	NA	NA	38	NA
Survey, APDC and PBS	11	7	2	7	3	23	3	7	7	NA
Survey and APDC and MBS	12	3	4	2	NA	3	NA	NA	9	NA
Survey and ACD	NA	NA	NA	NA	NA	NA	NA	NA	NA	29
APDC and ACD	NA	NA	NA	NA	NA	NA	NA	NA	NA	31
Survey, APDC and ACD	NA	NA	NA	NA	NA	NA	NA	NA	NA	19

^a^ Eligible women from the Australian Longitudinal Study on Women’s Health (born 1973–78 or 1989–95).

CKD = Chronic Kidney Disease; Arthrop = Autoinflammatory arthropathies and connective tissue disease; IBD = Inflammatory bowel disease; MS = Multiple Sclerosis.

APDC = Admitted Patient Data Collection; PBS = Pharmaceuticals Benefits Scheme; MBS = Medicare Benefits Schedule; ACD = Australian Cancer Database.

### Prevalence of chronic disease based on data source

**Fig 3:**
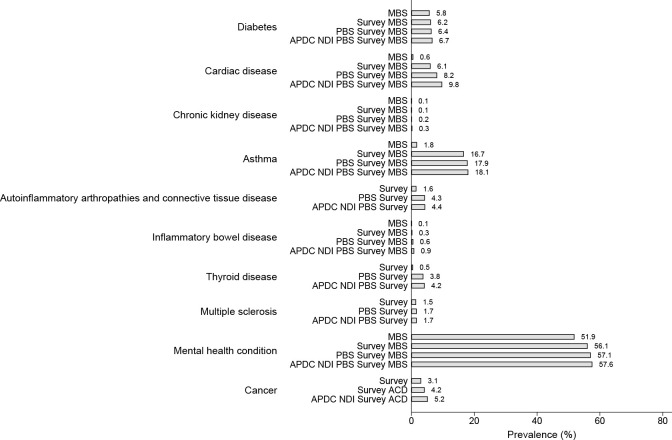
Prevalence of chronic conditions by June 2020 among Australian women aged 42–47 years (accounting for the progressive inclusion of additional data sources according to the typical timing of diagnosis and management for each condition).

**Fig 4:**
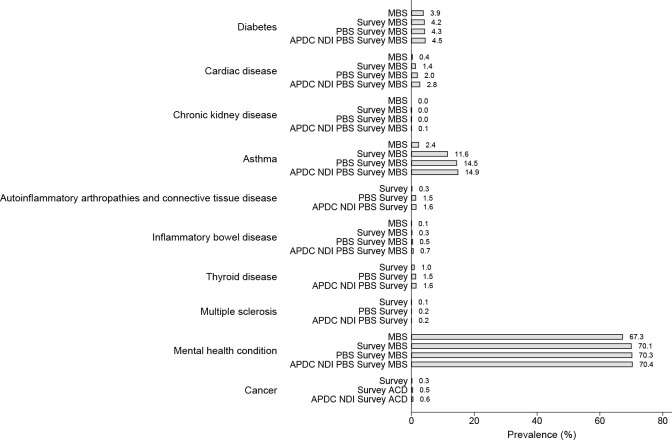
Prevalence of chronic conditions by June 2020 among Australian women aged 25–31 (accounting for the progressive inclusion of additional data sources according to the typical timing of diagnosis and management for each condition.

Figs 3 and 4 show the yield gain in chronic disease prevalence for each of the cohorts with the use of additional data sources (based on the typical clinical disease course) over the observation period. The highest yield gain for asthma was the addition of survey self-report to the MBS for asthma (increasing from 2.4% to 11.6% for the 1989–95 cohort and 1.8% to 16.7% for the 1973–78 cohort). It also provided the highest yield gain for cardiac disease (increasing from 0.4% to 1.4% for the 1989–95 cohort and 0.6% to 6.1% for the 1973–78 cohort). Gain for asthma and cardiac disease was modest with only a small yield gained by the addition of the PBS and APDC. The highest yield gain for autoinflammatory arthropathies and connective tissue disease included the addition of PBS to survey data with the prevalence increasing from 0.3% to 1.5% for the 1989–95 cohort and 1.6% to 4.3% for the 1973–78 cohort. Only a 0.1% increase in prevalence was demonstrated across both cohorts with the addition of the APDC/NDI datasets. A similar pattern was identified for thyroid disease. Further, the addition of any dataset to the MBS only resulted in small gains in the lifetime prevalence of mental health conditions. Rare conditions such as inflammatory bowel disease, cancer and chronic kidney disease benefitted from the addition of all available data sources with the prevalence incrementally increasing with the addition of each data source.

### Prevalence of chronic disease over time

Table 4 shows the cumulative prevalence for the 1989–95 and 1973–78 cohorts by June 2020 when the women were aged 25–31 years and 42–47 years respectively using the developed algorithms. All conditions had a higher lifetime prevalence among the 1973–78 cohort compared to the 1989–95 cohort except for mental health conditions which was estimated at 70.4% for the 1989–95 cohort and 57.6% for the 1973–78 cohort. Chronic kidney disease was rare across both cohorts with less than 0.5% of women reporting the condition. The cumulative prevalence of inflammatory bowel disease however was similar in both cohorts with 0.9% of women from the 1973–78 cohort and 0.7% of women from the 1989–95 cohort reporting these conditions. Overall, a slightly higher proportion of women from the 1989–95 cohort reported having at least one chronic health condition (75.2% vs 71.3%), although a higher proportion of women from the 1973–78 reported multimorbidity (2 or more conditions; 27.4% vs 17.9%).

**Table 4:** Cumulative prevalence of chronic conditions by June 2020 for the 1973–78 (when aged 42–47 years) and 1989–95 (when aged 25–31 years) cohorts of the Australian Longitudinal Study on Women’s Health using the standard and modified algorithm^a^.

	1973–78 cohort (N = 13,501)				1989–95 cohort (N = 16,964)			
Chronic condition	Standard Algorithm		Modified Algorithm		Standard Algorithm		Modified Algorithm	
	**N**	**% (95% CI)**	**N**	**% (95% CI)**	**N**	**% (95% CI)**	**N**	**% (95% CI)**
Diabetes	910	6.7 (6.3, 7.2)	982	7.3 (6.8, 7.7)	760	4.5 (4.2, 4.8)	794	4.7 (4.4, 5.0)
Cardiac disease	1324	9.8 (9.3, 10.3)	1610	11.9 (11.4, 12.5)	481	2.8 (2.6, 3.1)	566	3.3 (3.1, 3.6)
CKD	44	0.3 (0.2, 0.4)	47	0.3 (0.3, 0.5)	12	0.1 (0.0, 0.1)	14	0.1 (0.0, 0.1)
Asthma	2444	18.1 (17.4, 18.8)	2945	21.8 (21.1, 22.5)	2530	14.9 (14.4, 15.5)	3048	18.0 (17.4, 18.6)
Arthrop	600	4.4 (4.1, 4.8)	823	6.1 (5.7, 6.5)	266	1.6 (1.4, 1.8)	270	1.6 (1.4, 1.8)
IBD	118	0.9 (0.7, 1.0)	122	0.9 (0.8, 1.1)	118	0.7 (0.6, 0.8)	122	0.7 (0.6, 0.9)
Thyroid disease	572	4.2 (3.9, 4.6)	577	4.3 (3.9, 4.6)	268	1.6 (1.4, 1.8)	481	2.8 (2.6, 3.1)
MS	229	1.7 (1.5, 1.9)	394	2.9 (2.6, 3.2)	31	0.2 (0.1, 0.3)	75	0.4 (0.3, 0.6)
Mental health	7777	57.6 (56.8, 58.4)	7948	58.9 (58.0, 59.7)	11935	70.4 (69.7, 71.0)	12173	71.8 (71.1, 72.4)
Cancer	708	5.2 (4.9, 5.6)	1057	7.8 (7.4, 8.3)	101	0.6 (0.5, 0.7)	126	0.7 (0.6, 0.9)
^b^Cardiometabolic diseases	2032	15.1 (14.5, 15.7)	2337	17.3 (16.7, 18.0)	1195	7.0 (6.7, 7.4)	1306	7.7 (7.3, 8.1)
^c^Autoinflammatory diseases	1428	10.6 (10.1, 11.1)	1776	13.2 (12.6, 13.7)	656	3.9 (3.6, 4.2)	911	5.4 (5.0, 5.7)
Total number of conditions	**N**	**%**	**N**	**%**	**N**	**%**	**N**	**%**
0	3860	28.6	3440	25.5	4205	24.8	3867	22.8
1	5903	43.7	5537	41.0	9723	57.3	9397	55.4
2	2679	19.8	3086	22.9	2405	14.2	2930	17.3
3	817	6.0	1051	7.8	564	3.3	678	4.0
4 or more	242	1.8	387	2.9	67	0.4	92	0.5

^a^ The standard case ascertainment algorithm requires two of more self-reports through different surveys; the modified algorithm allows for women to self-report once only if it was first reported at their last returned survey.

^b^ Cardiometabolic disease is the combined prevalence of diabetes, cardiac disease and chronic kidney disease.

^c^ Autoinflammatory disease is the combined prevalence of autoinflammatory arthropathies and connective tissue disease, inflammatory bowel disease, multiple sclerosis and thyroid disease.

CKD = Chronic Kidney Disease; Arthrop = Autoinflammatory arthropathies and connective tissue disease; IBD = Inflammatory bowel disease; MS = Multiple Sclerosis.

**Fig 5:**
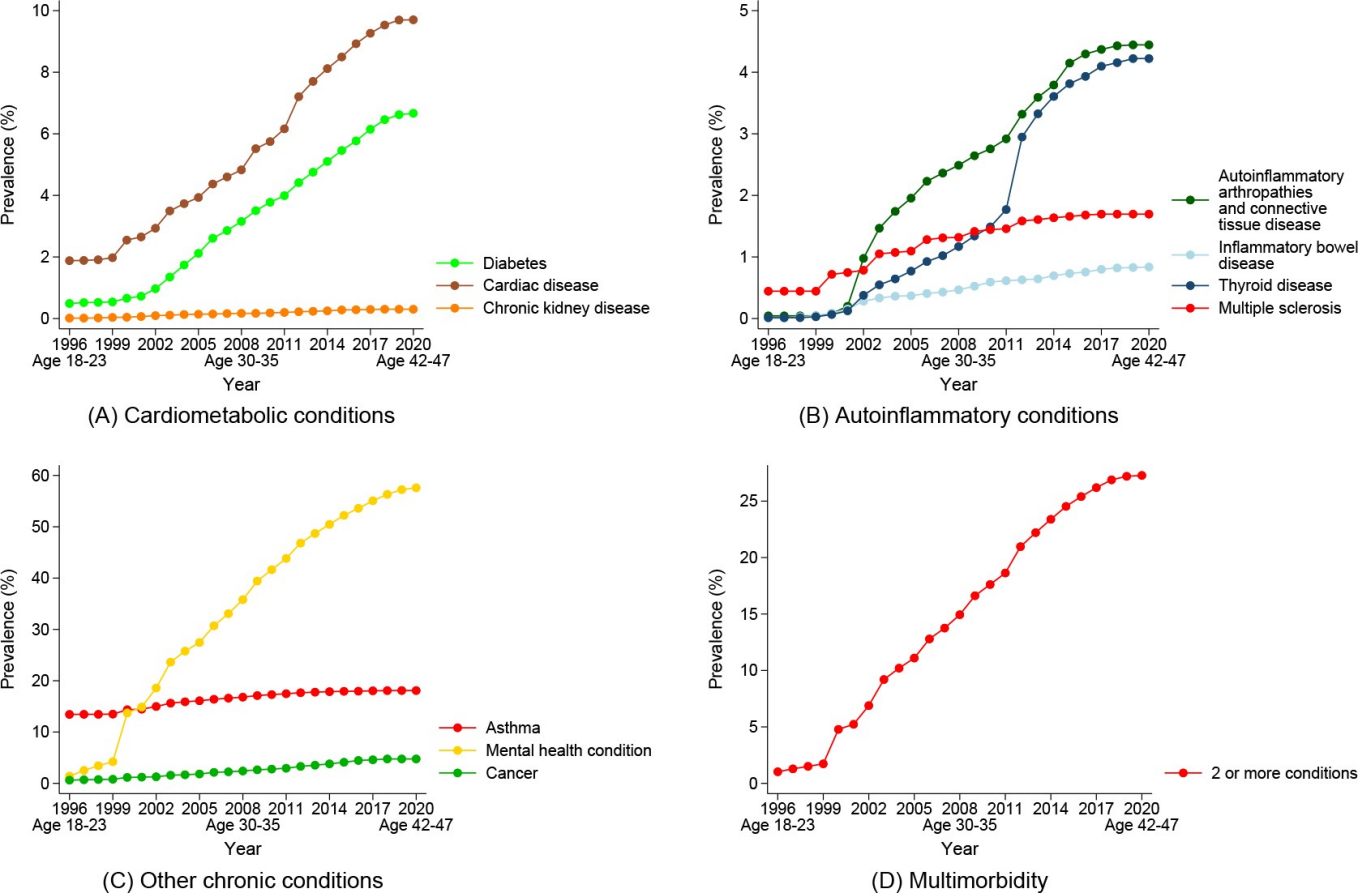
Prevalence of chronic conditions over time for women born 1973–78 from the Australian Longitudinal Study on Women’s Health.

**Fig 6:**
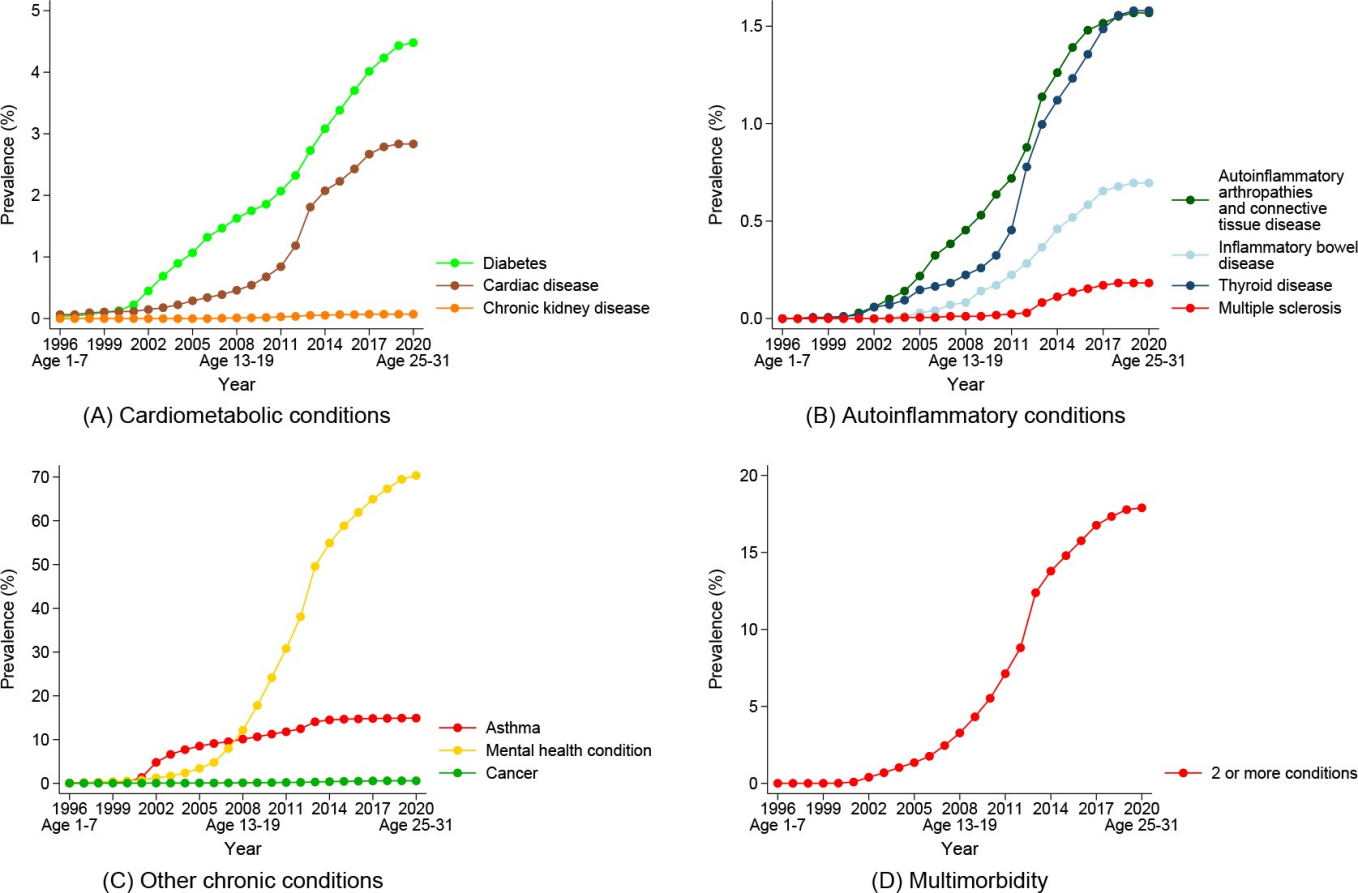
Prevalence of chronic conditions over time for women born 1989–95 from the Australian Longitudinal Study on Women’s Health.

Examining the prevalence of each of the conditions over the observation period, there was a consistent upward trend for cardiac disease, diabetes, and autoinflammatory arthropathies and connective tissue disease across both cohorts (see Figs 5 and 6). There were sharp increases in the prevalence of thyroid disease around 2012/13 in both cohorts, although this steep increase persisted for longer among the 1989–95 cohort. The trend in the prevalence of asthma was stable over time, with a slight increase beginning in 2002 for the 1989–95 cohort when they were aged 7–13 years. An increase in the identification of mental health conditions were noted in 1999/2000 for the 1973–78 cohort when items regarding diagnosed depression and anxiety were introduced in the ALSWH Survey. Mental health conditions were identified much earlier among the 1989–95 cohort, with the prevalence beginning to increase rapidly in approximately 2008 (when aged 13–19 years).

Comparing chronic disease prevalence between the cohorts when women were approximately the same age (women in the 1989–95 cohort were aged 25–31 in 2020, while women in the 1973–78 cohort were aged 25–30 in 2003), women from the 1989–95 cohort had more than double the prevalence of diabetes to women from the 1973–78 cohort (4.5% vs. 1.3%), with the difference between cohorts in terms of lifetime mental health conditions substantially higher (70.4% vs. 23.6%). Women from the 1989–95 cohort had lower prevalence of multiple sclerosis and cancer compared to women from the 1973–78 cohort at the same age. Prevalence rates were similar between the cohorts for asthma, autoinflammatory arthropathies and connective tissue disease, and chronic kidney disease. However, women from the 1989–95 cohort had higher prevalence of inflammatory bowel disease. In terms of multimorbidity, a consistent upward trend was identified for both cohorts, although this trend appears to be more pronounced in the 1989–95 cohort. The prevalence of multimorbidity by age 25–31 years for the 1989–95 cohort was 17.9%, while multimorbidity for the 1973–78 cohort was only 9.1% at the same age.

### Sensitivity analysis

Table 4 also compares the prevalence rates obtained using the standard algorithms and the modified algorithms for each condition. The standard case ascertainment algorithm requires two or more self-reports through different surveys, whereas the modified algorithm also allows for women to self-report once only if it was first reported at their last returned survey. We observed an increase in the prevalence in all conditions in both cohorts, but this increase ranged from an additional two cases for chronic kidney disease in the 1989–95 cohort, to an increase of 518 asthma cases in the 1989–95 cohort, which increased the prevalence from 14.9% to 18.0%. A similar increase in the prevalence of asthma was observed in the 1973–78 cohort. Overall, the modified algorithm resulted in a negligible increase in the prevalence rates for inflammatory bowel disease and chronic kidney disease and prevalence increases of less than 4% for the other conditions.

## Discussion

### Main findings

This study estimated the prevalence of ten chronic diseases associated with adverse maternal and foetal outcomes among two cohorts of women of reproductive age. These chronic conditions are also notable in terms of national health priority areas identified by the Australian government. We developed chronic disease-specific algorithms using multiple data sources, finding the contribution of each data source used for case ascertainment varied substantially based on the condition of interest. With the exception of mental health conditions, the lifetime prevalence of each of the chronic conditions was higher among women from the 1973–78 cohort. However, when focused on point prevalence at similar ages, the chronic disease trend for women in the 1989–95 cohort is substantially higher, particularly with respect to diabetes, cardiac disease, thyroid disease and multimorbidity. These findings have implications for the delivery of chronic disease preventive interventions and complicate the provision of contraception and family planning services.

### Interpretation

Given the lack of a gold standard in assessing agreement between data sources, we evaluated concordance using a simplified method that was not biased by the prevalence of the condition, which can be problematic particularly when low prevalence chronic diseases are concerned [12]. Using this approach, we found relatively low concordance between data sources across the two cohorts. With the exception of asthma and multiple sclerosis, a higher proportion of women were identified through the use of administrative data compared to survey data, with only a few conditions identified across the majority of sources (e.g., mental health conditions). Low concordance between self-report and administrative data is supported by others [10, 12, 13, 16]. Similar to previous research, we found that conditions that have greater contact with the healthcare system for ongoing disease management were more likely to be ascertained through administrative data sources [12, 22, 43]. In our study, 70% of diabetes cases were identified through a single data source, with the majority being identified by MBS items related to the Diabetes Annual Cycle of Care (a scheme introduced in 2001 which provides general practitioners with incentives for early diagnosis, monitoring, and effective management of diabetes). Although previous research examining the agreement between administrative data and surveys involving older populations (including the ALSWH) have largely found good agreement between survey and hospital data [8, 12, 43], our findings demonstrate that inpatient records do not adequately capture diabetes when focused on women of reproductive age.

We found that concordance between any two datasets was low (<20%) and only 14% of cases were identified through both survey and hospital data. Given the age of the women and disease ascertainment in administrative data sources being inextricably intertwined with disease severity [12], this suggests that diabetes in this cohort is relatively well-managed in primary care. It is important to note however, that there is the potential for diabetes cases to have been over-identified in the MBS given that we used the item code for HbA1c monitoring in established diabetes in our algorithm which may be incorrectly coded if used for screening purposes. Comino and colleagues found that sensitivity for this item number varied depending on the number of tests included in the algorithm, with highest sensitivity being for two or more tests over their five-year observation period (74%) [44]. We accounted for this potential by requiring at least two tests within a calendar year for inclusion as this is in line with minimum management guidelines for monitoring diabetes. We also increased the potential for inclusion through this data source by including other allied health-related Diabetes Annual Cycle of Care items in the algorithm; and as such, those that had HbA1c as their only indicator was minimal.

In contrast to diabetes where 82% of cases had an indication in the MBS, only 13% of asthma cases were identified through similar asthma-related disease management items (Asthma Cycle of Care), with the majority identified in either the survey or PBS. Studies in other countries with universal health care systems that have employed disease-related algorithms have found that a sensitive algorithm for identifying asthma cases from administrative data over a period of five years can be obtained by using pharmacy records either alone or in combination with hospital and physician billing claims, regardless of age (although these were obtained at a province population-level as opposed to national level) [19]. In contrast, Fortin found that 87% of asthma cases in Quebec were obtained via self-report compared to 43% physician claims over a two year look back period [13]. Here, with over 20 years of MBS data (including complete capture for specific asthma management items under its various iterations), we found that physician claims only contributed a small amount to the overall prevalence of asthma and had low concordance with all other data sources. Low use of the asthma cycle of care management plan (and its predecessor, Asthma 3+ visits) in Australia is not new and has been demonstrated across all age groups, particularly for young women [6, 45]. Despite conjecture in the literature regarding the validity of self-report asthma status, high reliability of self-reported history of asthma has been found over five years across sociodemographic and health-related factors, particularly when participants were asked about lifetime asthma diagnoses. This approach has been found to increase the capture of mild cases [46]. We recommend any multi-source algorithm aimed at ascertaining asthma among women of reproductive age include both survey and medication data.

Mental health conditions had the highest concordance between administrative data and survey data with 64% of cases identified in the survey and at least one of the administrative datasets, with 93% of cases indicated through the MBS data. MBS items were introduced in 2006 under the Better Access Scheme for specified mental health conditions and included services delivered by allied mental health care practitioners, clinical psychologists, occupational therapists and psychiatrists (as referred by a general practitioner under a mental health plan). Women from the 1989–95 cohort had substantially higher lifetime reports of mental health conditions compared to women from the 1973–78 cohort with self-reported diagnosed conditions and hospital data providing little in terms of overall yield gain in prevalence. Point prevalence for these conditions prior to 2006 (when the Better Access Scheme was implemented), however, needs to be viewed with caution as self-report survey items varied in terms of mental health specificity. In the 1973–78 cohort an increase in lifetime mental health conditions were noted between 1999 and 2000 with the introduction of the depression and anxiety items to the ALSWH surveys while mental health items for the 1989–95 cohort were more inclusive from the baseline survey in 2012/13 (e.g., containing items on eating and bipolar disorders). While higher prevalence may be attributed to increased detection and treatment opportunities as well as reduced stigma surrounding mental health conditions, there is evidence to suggest that mental health conditions are increasing among young people and that females are twice as likely to be diagnosed compared to males [47]. Our lifetime prevalence rates across both cohorts far exceed national prevalence estimates based solely on survey data and point to the need for increased focus on both treatment and prevention strategies [48]. Given that a high proportion of lifetime mental health conditions were diagnosed by early adulthood for the 1989–95 cohort, increased resources particularly in the child and adolescent space are required to meet future mental health challenges. This is particularly important given the evidence indicating that a woman’s prior mental health history plays a role in increased vulnerability to mental health issues in the perinatal period as well as the emerging evidence regarding the mental health impact associated with COVID-19 restrictions [49, 50].

It is important to note that conditions that were not listed in the main survey and required the participant to volunteer disease information as a free-text response had lower identification through surveys compared to administrative data, with the exception of multiple sclerosis. As a result, we have potentially under-reported the prevalence of these conditions. Given that conditions such as rheumatoid arthritis and multiple sclerosis are of a relapsing and remitting nature, adherence to medications might also vary and would be problematic with only a short observation window. However, we had a long observation period in which to examine medication use, allowing for the natural disease course to be identified (particularly with respect to the 1973–78 cohort). Further, for medications that have multiple indications (such as with some cardiac and inflammatory conditions), inclusion of medication data that was verified through another source increased the precision of the PBS notifications. This finding is supported by Kim and colleagues who found that diagnosis code-based algorithms alone are not sufficient to accurately identify rheumatoid arthritis cases through administrative data and that algorithms that link pharmacy claims for disease-modifying anti-rheumatic drugs can improve positive predictive value [51]. Therefore, while medication data appears to play an important role in the ascertainment of a number of chronic diseases, the use of multiple datasets are recommended to overcome the barriers associated with medications that have multiple indications.

Overall, for women of reproductive age, hospital data only provided small gains in yield for the majority of conditions and its relevance to the condition was based on the seriousness of the disease. Inclusion of hospital data was found to be important for chronic kidney disease, inflammatory bowel disease and cancer (particularly as the ACD was only available until 2016). Around 40% of cardiac cases were also identified in the hospital data. Given that we combined hypertension (which is often managed in primary care) with other forms of cardiac disease, this would have impacted on the concordance between the data sources [8]. Chronic disease indications in the hospital data may also be impacted by reporting practices with not all chronic diseases being recorded during an episode of care as well as between-hospital variation [52]. In particular, listing of additional diagnoses in medical records has only been available since 2015. Prior to this time, only diagnoses related to the specific episode of care were coded.

In our study, we demonstrated that depending on the chronic condition, use of any one source alone may dramatically under-estimate cases in the sample. Use of multiple data sources and application of an algorithm for case ascertainment for women of reproductive age has clear advantages, given the low prevalence of some diseases. Given the strengths and limitations associated with each particular data source, this approach to providing population-level estimates is suitable for chronic disease surveillance and health care planning. Where a researcher does not have access to multiple data sources, it is important that they are overt regarding the purposes of the study, select the data sources that are likely to provide the highest yield, and clearly identify the limitations of the data sources used. Rector and colleagues indicate that when high sensitivity is of primary interest, adding diagnostic or pharmacy claims or using a longer period of time to capture claims that list a diagnosis for a chronic condition or medication is preferred [22]. Our findings support this assumption to some extent; however, the value of population-level survey data cannot be overlooked and fills in the gaps for conditions where pharmacy data would not identify people with particular conditions (e.g., type 2 diabetes and hypertension being managed by diet or early-stage multiple sclerosis being managed by physical therapies). This is particularly important for our sample where multiple sclerosis was more likely to be identified in the survey data than in either the medication or hospital data. This contrasts with previous research which suggested that an algorithm that required only one hospital admission or one physician encounter resulted in high sensitivity [21]. While we did not have specific physician-based items for comparability, in our sample the use of hospital data and medication data was found to be low despite a long observation period. We also echo the sentiments of Lujic and colleagues who indicated that not only morbidity, but also multimorbidity prevalence will vary depending on which data sources are used [11]. It is important to note that regardless of the data sources used, there will be missed cases as well as the potential for under- or over-reporting of chronic diseases and it is the responsibility of the researcher to identify and clearly describe any biases these data sources introduce into their findings. Our sensitivity analysis demonstrated that for the data sources we used, this was relatively low, but was condition dependent. Results from observational studies involving women of reproductive age must therefore be considered in light of the data sources used for ascertainment. In addition, these findings are specific to the data sources available in Australia. Our findings would need to be replicated in other countries using available data sources.

### Strengths and limitations

Ascertaining chronic disease cases using two nationally representative birth cohorts of women of reproductive age is a particular strength of this study. This allowed us to not only estimate the prevalence of key chronic diseases but also provided some insight into the generational differences in chronic disease prevalence. Multiple record linkage to population-level survey data over long periods also allowed us to ascertain both congenital and acquired forms of disease (e.g., congenital heart disease). Other studies assessing concordance between data sources have often used a limited number of administrative databases and up to a five-year window [11, 12, 19, 53]. Given the age of these women, ascertainment using these processes would fail to capture diseases acquired in childhood. Further, the number of data sources examined allowed us to capture chronic diseases along the clinical disease course, with less severe cases (where they might be managed by non-pharmacological approaches or be asymptomatic) being captured in the surveys. This reduced the bias associated with ascertaining cases purely from one data source. Coupled with this, the employment of disease specific algorithms in consultation with clinical and content experts allowed us to provide a more complete, and reliable picture of disease prevalence among women of reproductive age (particularly with the inclusion of non-descript medications in the PBS data when an additional source was able to corroborate the use for that specific condition).

Despite this, our study must be considered in light of a few limitations. Firstly, we based our assessment on a defined set of chronic diseases. In doing so, this impacted on the overall prevalence estimate of having at least one chronic health condition and also multimorbidity. Other high prevalence conditions, such as endometriosis and polycystic ovary syndrome, were not assessed in this study as this paper served to estimate the prevalence of chronic conditions associated with poor maternal and foetal outcomes and which would benefit from preconception contraceptive counselling interventions. However, the conditions we did assess were national health priorities and largely managed in primary care, which accounts for the significant chronic disease burden. Although the development of considered algorithms for each condition was a strength of the study, we did not assess multiple algorithms to understand which specific algorithm had the best validity. The purpose here was to assess the concordance between data sources and to understand the yield gain by adding data sources in order to provide researchers with an understanding of who may or may not be captured by the data sources they are using when the focus is on women of reproductive age. There is also the possibility that despite our best efforts, some cases may have been attributed to false negatives. While we minimised the impact of this through a conservative algorithm (e.g., having to have indications in two surveys or two prescriptions per calendar year across two years) and consultation with clinicians who treat women with these conditions, there may have been a minority of women who were provided some prescriptions ‘off label’. In addition, as we developed a conservative algorithm, there may be women who were classified as not having the disease due to cohort attrition, particularly those in poorer health (although we had coverage through the administrative data). We addressed this issue by including women who had one self-report indication but were lost-to-follow-up as cases in our sensitivity analyses. Given that younger women in the ALSWH cohort have been found to be transient in their completion of surveys [54, 55], this is expected to have only contributed to a small increase in prevalence, although we acknowledge that the use of self-report requires the participant to have knowledge of, and a willingness to report, their disease. Importantly, there are inherent biases associated with any data source, with diseases identified through administrative data likely to involve more severe disease or disease activity. In addition, one of the challenges associated with combining definitions based on both administrative and survey data is around the calculation of disease prevalence and incidence. While there is an encounter-based anchor present in administrative data around when healthcare was sought, there are difficulties around ascertaining the exact timing of disease diagnosis in self-report data. This makes it difficult to calculate disease incidence. Use of a chronic disease algorithm that includes self-report survey data assists with the ascertainment of mild cases of disease and is more appropriate for ascertaining lifetime cases of a specific disease at a certain point in time than incident cases. Finally, given that the survey checklist only focused on certain diseases, conditions such as inflammatory bowel disease, arthropathies and connective tissue disease and multiple sclerosis, relied on the participant reporting this as part of a free-text response. As such, these conditions may have been under-reported in the survey data (particularly those with only mild symptomatology).

### Implications

The increasing trend of chronic diseases among women of reproductive age, particularly with respect to cardiac disease, diabetes and autoinflammatory conditions among both cohorts is concerning for not only the purposes of chronic disease prevention but also for the provision of family planning. Importantly, when we compared women between cohorts at roughly the same age, we found that women from the 1989–95 cohort had higher prevalence of diabetes and cardiac disease (which was largely driven by hypertension), with the prevalence of both conditions continuously increasing with age across the observation period. A similar age-wise trend for diabetes has been demonstrated with the two oldest cohorts of the ALSWH [17]. While some of the increase in prevalence at earlier ages for these conditions may be attributed to improved diagnostic techniques and early detection, findings from the U.S. suggest that even among adults with regular primary care access, those aged 18–39 were unlikely to receive a hypertension diagnosis compared to adults at least 40 years old despite meeting criteria for diagnosis [56]. As a result, the prevalence trend for the 1989–95 cohort could be even higher with respect to cardiac disease, with a large proportion of cases of hypertension undiagnosed. As with mental health conditions, these findings highlight the need for strategic chronic disease interventions to be delivered during childhood and adolescence in order to curb the escalating chronic disease trend.

The increasing prevalence of chronic disease among women of reproductive age and particularly the trend for increasing multimorbidity has significant implications for the provision of contraceptive advice and reproductive planning. While there is an absence of high quality data, there is mounting evidence to suggest that women with chronic diseases experience unintended pregnancy at a higher rate than women without chronic disease [57]. However, for women with chronic disease, unintended pregnancies are associated with serious adverse maternal and perinatal outcomes, including congenital abnormalities, pre-term labour, spontaneous abortion, premature birth, low birthweight and foetal death [7]. Optimised preconception care and reproductive life planning among women with chronic diseases is therefore critical to the prevention of unintended pregnancies and reduction in pregnancy-related complications (particularly for women treated with medications with known teratogenic effects). Yet, it is suggested that these women report lower rates of contraceptive counselling and are less likely to use (or report inconsistent use of) contraception [58–60] despite clinical guidelines to assist clinicians with navigating the complexity of contraceptive use for some conditions (e.g. oestrogen-driven cancers) and chronic disease risk factors (e.g. obesity) [32]. Therefore, to prevent unintended pregnancy in this high-risk population, future research is required in understand how contraceptive use changes over time for women with chronic disease using population-level data.

## Conclusion

Population-level survey data linked to administrative data provides a powerful basis for chronic disease surveillance and allows for the examination of issues pertinent to women with chronic disease who are of reproductive age (e.g., in relation to family planning and pregnancy). Concordance of disease prevalence between multiple data sources in our study was observed to be low, signalling that the use of a single data source alone would be insufficient to capture the true prevalence of the disease. When unable to use multiple data sources for research involving women of reproductive age, we recommend that researchers be explicit about the limitations of the data sources used. Overall, using a multiple method approach we found that conditions such as diabetes, cardiac disease and inflammatory conditions are increasing across both cohorts but at higher rates among younger women. Lifetime mental health conditions were also found to be at alarmingly high levels for women of both cohorts. In order to reduce the increasing chronic disease and multimorbidity trend among women, strategic chronic disease interventions are required to be implemented in childhood and adolescence to ensure the long-term health of not only current but also future generations.

## Supporting information

S1 Table Administrative data sources used for chronic disease ascertainment (excluding Australian Cancer Database). (DOCX)

S2 Table ALSWH survey questions used for chronic disease ascertainment for the 1973–78 and 1989–95 cohorts. (DOCX)

S3 Table Case ascertainment algorithms. (DOCX)

S4 Table Baseline characteristics for eligible women (aged 18–23 years) from two cohorts of the Australian Longitudinal Study on Women’s Health^a^. (DOCX)
