# Supplementary material for: Increased chronic disease prevalence among the younger generation: Findings from a population-based data linkage study to inform chronic disease ascertainment among reproductive-aged Australian women
Source: PLoS One. 2021 Aug 18;16(8):e0254668. doi: 10.1371/journal.pone.0254668 (PMC8372972; doi:10.1371/journal.pone.0254668)
Supplement: S1 Table — (DOCX) [file pone.0254668.s001.docx]

**S1 Table. Administrative data sources used for chronic disease ascertainment (excluding Australian Cancer Database)**

| **Chronic disease** | **ICD-10-AM codes**  **(APDC/NDI)** | **ICD-9-AM codes**  **(APDC/NDI)** | **ATC codes***  **(PBS)** | **Item numbers**  **(MBS)** |
| --- | --- | --- | --- | --- |
| Diabetes | E10.x; E11.x; E13.x; E14.x | 250.x; 249.x | A10AB01; A10AB02; A10AB04; A10AB05; A10AB06; A10AB30; A10AC01; A10AC02; A10AC04; A10AC30; A10AD01; A10AD04; A10AD05; A10AD06; A10AD30; A10AE01; A10AE04; A10AE05; A10AE06; A10AE30; A10AF01; A10BA02; A10BB01; A10BB02; A10BB03; A10BB04; A10BB05; A10BB06; A10BB07; A10BB08; A10BB09; A10BB10; A10BB11; A10BB12; A10BB31; A10BC01; A10BD02; A10BD03; A10BD04; A10BD05; A10BD06; A10BD07; A10BD08; A10BD09; A10BD10; A10BD11; A10BD12; A10BD13; A10BD14; A10BD15; A10BD16; A10BD17; A10BD18; A10BD19; A10BD20; A10BD21; A10BD23; A10BD24; A10BD25; A10BF01; A10BG01; A10BG02; A10BG03; A10BH01; A10BH02; A10BH03; A10BH04; A10BH05; A10BH06; A10BJ01; A10BJ02; A10BJ03; A10BJ04; A10BJ05; A10BJ06; A10BK01; A10BK02; A10BK03; A10BK04; A10BX02; A10BX04; A10BX07; A10BX09; A10BX10; A10BX11; A10BX12; A10BX13; A10BX14 | 2517-2635; 259-264; 66551; 73840; 10915; 12325; 12326; 81100; 81105; 81110; 81115; 81120; 81125 |
| Cardiac disease | I10.x; I11.x-I13.x, I15.x I44.x – I45.x, I47.x-I49.x, T82.1, Z45.0, Z95.0; I42.x, I43.x, I50.x; I20.x- I25.x, I46.x; I05.x-I09.x; Q20.x-Q26.x; I51.0; I26.x-I28.x; I34.x-I38.x, A52.0, I05.x-I08.x, I09.1, I09.8, Q23.0-Q23.3, Z95.2-Z95.4 | 401.x; 402.x-405.x; 426.x, 427.0-427.4, 427.6-427.9, 996.01, 996.04, V45.0x, V53.3; 425.x, 428.x; 410.x- 414.x, 427.5; 393.x-398.x; 745.x-747.x, 429.71; 415.0, 415.1, 416.x, 417.x; 424.x, 746.x, V42.2, V43.3 | C02AB01; C02AB02; C02AC01; C02AC05; C02CA01; C02CA04; C02DB02; C02KX01; C02KX02; C02KX03; C02KX04; C02KX05; C02KX52; C03AA03; C03BA04; C03BA11; C02DC01; C07AG01; C07AG02; C08CA01; C08CA02; C08CA13; C08CA05; C08DA01; C08DB01; C09AA01; C09AA02; C09AA09; C09AA03; C09AA04; C09AA06; C09AA05; C09AA10; C09BA02; C09BA09; C09BA04; C09BA06; C09CA06; C09CA02; C09CA04; C09CA01; C09CA07; C09CA08; C09CA03; C09DA06; C09DA04; C09DA02; C09DA08; C09DA07; C09DA03; C09BB02; C09BB04; C09BB05; C09BB10; C09DB01; C09DB02; C09DX01; C09DB04; C01AA05; C01BA03; C01BA02; C01BA01; C01BB01; C01BB02; C01BC04; C01BD01; C01DA02; C01DA08; C01DA14; C01DX16; C01EB10; C01EB17; C02DD01; C03DA04; C03DA01; C03DB01; C03EA01; C07AA02; C07AA03; C07AA07; C07AA05; C07AB03; C07AB02; C07AB07; C07AB12; C08EX02; C01EB17; B01AC04; B01AC16; B01AC17; B01AC22; B01AC24; B01AC25; B01AC26; B01AD07; B01AD11; B01AE06; B01AE07; B01AA02; B01AA03; C09DX04; C01CE02; B01AF01; B01AF02 | 35304, 35305, 35310, 35335, 35338, 35341, 35344, 38300, 38303, 38306 38309, 38312; 38315, 38318; 38497-38504; 38281, 38470, 38473, 38209, 38212, 38350, 38353, 38356; 11627; 22060, 23010-24136; 21941; 38496-38509; 38512- 38518; 38600-38640; 38700- 38766; 38475-38493; 11718, 11721; 11719, 11720, 11721, 11725, 11726; 38365, 38368, 38654, 38371; 38384, 38387; 11727; 63385; 38358; 38275; 38390, 38393; 11627; 20560; 38653; 38700, 38703; 38706, 38709, 38712; 38715, 38718; 38721, 38724, 38727, 38730; 38739; 38742; 38745; 38748; 38754; 38757; 38760; 38763; 38766 |
| Chronic kidney disease (excluding complicated diabetes) | N18.x; D63.1; T82.4; T86.1; Z49.x; Z94.0; P96.0; Q27.1; Q27.2x; Q61.x-Q63.9 | 585.x; 285.21; 996.1; 996.81; V56.x; V42.0; 779.89; 753.1x; 753.22; 753.29; 753.4 | B03XA01; B03XA02; B03XA03; V03AE02; V03AE03; V03AE05; V03AE04; V03AE06; V03AE07; V03AE08; L04AA10; C09AA01, C09AA02, C09AA03, C09AA04, C09AA05, C09AA06, C09AA09, C09AA10; C09BA04, C09BA06, C09BA09; A11CC05; A12AA04; C09CA06; C09CA02; C09CA04; C09CA01; C09CA07; C09CA08; C09CA03; C09DA06; C09DA04; C09DA02; C09DA08; C09DA07; C09DA03; C09BA02; C09BA09; C09BA04; C09BA06 | 13100-13112; 36540, 36543; 36503, 36506, 36509; 66671 |
| Asthma | J45.x; J46.x | 493.x | R03AC02; R03AC03; R03AK06; R03AK07; R03AK10; R03BA01; R03BA02; R03BA05; R03BA08; R03BA09; R03BB01; R03BB04; R03BC01; R03BC03; R03CC02; R03CC03; R03DA04; R03DC03; R03DX05; L04AC06; R03DX09; R03DX10 | 2546-2559; 2664-2677 |
| Autoinflammatory arthropathies and connective tissue disorders | M05.x-M06.x; M07.x; L40.5x; M08.x, M09.x; M45.x; M32.x, M34.x-M35.3; M09.1x- M09.2x | 714.x; 713.x; 714.x; 696.0; 714.x; 720.0; 710.x, 711.2x, 725 | L04AA11; L04AB01; L04AB02; L04AA12; L04AB04; L04AA17; L04AB05; L04AB06; L04AX01; L04AC13; L04AC07; L04AC03, L04AA14; L04AC05; L04AC10; A07EC01; P01BA02; M01AB01, M01AB02, M01AB05, M01AB55; M01AC01, M01AC06, M01AC02; M01AE01, M01AE02, M01AE03; M01AE11, M01AE51; M01AG01; M01AH01, M01AH02, M01AH05, M01AH06; M01CB01; M01CB03; M01CC01; L04AA24; L04AA13; L04AA26; L04AA29; L04AA37; L04AA06; L01XC02; L04AA01; L04AD01; L04AA05; L04AD02; L01AA01; L01BA01; L04AX03; | ---- |
| Inflammatory bowel disease | K50.x; K51.x | 555.x; 556.x | A07EC01; A07EC02, A07EC03, A07EC04; L04AA33; A07EA06; A07EA02; L04AB02 L04AA12; L04AB04; L04AB06; L04AC05; L04AX01; L01BA01; L04AX03; L01BB02; L04AA01; L04AD01; L04AA05; L04AD02; M04AA01 | 63744; 63741; 63746; 63743; 63747 |
| Thyroid disease | E00.x-E03.x; E05.x; E06.x | 240.9, 243, 244.x, 246.1, 246.8; 242.x, 376.21, 376.22; 245.x | H03AA01; H03AA02; H03AA03; H03BA01; H03BA02; H03BA03; H03BB01; H03BB02; H03BB52; H03BC01; H03BX01; H03BX02 | ---- |
| Multiple sclerosis | G35 | 340 | L04AA23, L04AA27; L04AA31; L01XC04; L04AA34; L04AA36; N07XX09; L04AX07; L03AB07; L03AB08; L03AB13; L03AX13; L01BB04; L01DB07; N07XX07; L04AX01; L01AA01; L01BA01; L04AX03; L04AC01 | ---- |
| Mental health conditions | F30.x-F39.x; F20.x-F28.x; F40-F48; F60.x; F50.x; | 296.x, 298.0; 301.1x; 300.4; 295.x, 297.0-298.9, 301.22; 300.x; 306.x; 308.x-311; 301.x; 307.1; 307.5x; | N06AF03; N06AF04; N06AB04; N06AB10; N06AB03; N06AB08; N06AB05; N06AB06; N06AA09; N06AA04; N06AA16; N06AA12; N06AA02; N06AA10; N06AA01; N06AA06; N06AX23; N06AX21; N06AX17; N06AX16; N06AX03; N06AX11; N06AX18; N06AX22; N06AX26; N06AX06; N06AG02; N05AA01; N05AA06; N05AA03; N05AB06; N05AB08; N05AB10; N05AB02; N05AB04; N05AC01; N05AC02; N05AC03; N05AD01; N05AD08; N05AD03; N05AD06; N05AD07; N05AE04; N05AE05; N05AE02; N05AF01; N05AF03; N05AF04; N05AF05; N05AG01; N05AG02; N05AG03; N05AH01; N05AH02; N05AH03; N05AH04; N05AH05; N05AH06; N05AL01; N05AL02; N05AL03; N05AL05; N05AL06; N05AN01; N05AX07; N05AX08; N05AX12; N05AX13; N05AX14; N05AX15; N05AX16; N05BA01; N05BA02; N05BA03; N05BA04; N05BA05; N05BA06; N05BA07; N05BA08; N05BA09; N05BA10; N05BA11; N05BA12; N05BA13; N05BA14; N05BA15; N05BA16; N05BA17; N05BA18; N05BA19; N05BA21; N05BA22; N05BA23; N05BA24; N05BA56; N05BB01; N05BB51; N05BC01; N05BC03; N05BC05; N05BC51; N05BD01; N05BE01; N05BX03; N06CA01; N06CA02; N06CA03; N03AX09; N03AF01; N03AG01; N03AE01 | 272-282, 283, 285, 286, 287, 371, 372; 2700 – 2719; 2721, 2723, 2725, 2727, 2729, 2731; 2574, 2575, 2577, 2578; 2704, 2705, 2707, 2708; 291, 293, 300, 302, 304, 306, 308, 310, 312, 314, 316, 318, 319; 320, 322, 324, 326, 328; 330, 332, 334, 336, 338; 342, 344, 346; 2121, 2150, 2196; 10956; 10968; 80000, 80001, 80005, 80010, 80011, 80015, 80020, 80021; 80100, 80101, 80105, 80110, 80111, 80115, 80120, 80121, 80125, 80126, 80130, 80135, 80136, 80140, 80145, 80146, 80150, 80151, 80155, 80160, 80161, 80165, 80170, 80171; 14224, 20104; 288, 353-370; 855-866; 81355; 81325 |
| Cancer | C00-C97 | 140.x-174.x; 176.x- 202.x | ---- | ---- |

* Note: ICD=International Classification of Diseases; MBS=Medicare Benefits Schedule; PBS=Pharmaceuticals Benefits Scheme; APDC=Admitted Patient Data Collection; NDI=National Death Index. Aspirin and corticosteroids were excluded due to their generic role of reducing inflammation.
