# Supplementary material for: Increased chronic disease prevalence among the younger generation: Findings from a population-based data linkage study to inform chronic disease ascertainment among reproductive-aged Australian women
Source: PLoS One. 2021 Aug 18;16(8):e0254668. doi: 10.1371/journal.pone.0254668 (PMC8372972; doi:10.1371/journal.pone.0254668)
Supplement: S2 Table — (DOCX) [file pone.0254668.s002.docx]

**S2 Table. ALSWH survey questions used for chronic disease ascertainment for the 1973-78 and 1989-95 cohorts**

**ALSWH 1973-78 cohort**

| **Survey question** | **S1** | **S2** | **S3** | **S4** | **S5** | **S6** | **S7** | **S8** |
| --- | --- | --- | --- | --- | --- | --- | --- | --- |
| **Diabetes** |  |  |  |  |  |  |  |  |
| Have you ever been told by a doctor that you have: Diabetes (high blood sugar) | **✓** |  |  |  |  |  |  |  |
| Have you ever been told by a doctor that you have: Insulin dependent (Type I) diabetes Yes, in the last 4 years |  | **✓** |  |  |  |  |  |  |
| Have you ever been told by a doctor that you have: Non-insulin dependent (Type II) diabetes Yes, in the last 4 years |  | **✓** |  |  |  |  |  |  |
| In the past three years, have you been diagnosed or treated for: Insulin dependent (type 1) diabetes |  |  | **✓** | **✓** | **✓** | **✓** | **✓** | **✓** |
| In the past three years, have you been diagnosed or treated for: non-Insulin dependent (type 2) diabetes |  |  | **✓** | **✓** | **✓** | **✓** | **✓** | **✓** |
| **Cardiac disease** |  |  |  |  |  |  |  |  |
| Have you ever been told by a doctor that you have: Heart disease | **✓** |  |  |  |  |  |  |  |
| In the past three years, have you been diagnosed or treated for: Heart disease (including heart attack, angina) |  |  | **✓** | **✓** | **✓** | **✓** | **✓** | **✓** |
| Have you ever been told by a doctor that you have: Hypertension (high blood pressure) | **✓** |  |  |  |  |  |  |  |
| Have you ever been told by a doctor that you have: Hypertension (high blood pressure) other than during pregnancy Yes, in the last 4 years |  | **✓** |  |  |  |  |  |  |
| In the last three years, have you been diagnosed or treated for: Hypertension (high blood pressure) other than during pregnancy |  |  | **✓** | **✓** |  |  |  |  |
| In the past three years, have you been diagnosed or treated for: High blood pressure (hypertension) |  |  |  |  | **✓** | **✓** | **✓** | **✓** |
| **Asthma** |  |  |  |  |  |  |  |  |
| Have you ever been told by a doctor that you have: Asthma | **✓** |  |  |  |  |  |  |  |
| Have you ever been told by a doctor that you have: Asthma Yes, in the last 4 years |  | **✓** |  |  |  |  |  |  |
| In the past three years, have you been diagnosed or treated for: Asthma |  |  | **✓** | **✓** | **✓** | **✓** | **✓** | **✓** |
| **Autoinflammatory arthropathies** |  |  |  |  |  |  |  |  |
| Have you ever been diagnosed with or treated for: Rheumatoid arthritis |  |  |  |  |  |  | **✓** |  |
| In the last 3 years, have you been diagnosed with or treated for rheumatoid arthritis |  |  |  |  |  |  |  | **✓** |
| Have you ever been diagnosed with or treated for: Other arthritis |  |  |  |  |  |  | **✓** |  |
| In the last 3 years, have you been diagnosed with or treated for other arthritis |  |  |  |  |  |  |  | **✓** |
| **Mental health conditions** |  |  |  |  |  |  |  |  |
| Have you ever been told by a doctor that you have: Depression (not postnatal) Yes, in the last 4 years |  | **✓** |  |  |  |  |  |  |
| Have you ever been told by a doctor that you have: Depression (not postnatal) Yes, more than 4 years ago |  | **✓** |  |  |  |  |  |  |
| In the past three years, have you been diagnosed or treated for: Depression (not postnatal) |  |  | **✓** | **✓** | **✓** | **✓** | **✓** | **✓** |
| Have you ever been told by a doctor that you have: Anxiety disorder Yes, in the last 4 years |  | **✓** |  |  |  |  |  |  |
| Have you ever been told by a doctor that you have: Anxiety disorder Yes, more than 4 years ago |  | **✓** |  |  |  |  |  |  |
| In the past three years, have you been diagnosed or treated for: Anxiety/nervous disorder |  |  | **✓** | **✓** | **✓** | **✓** | **✓** | **✓** |
| In the last three years, have you been diagnosed or treated for: Post traumatic stress disorder (PTSD) |  |  |  |  |  |  | **✓** | **✓** |
| In the last three years, have you been diagnosed or treated for: Bipolar disorder |  |  |  |  |  |  | **✓** | **✓** |
| In the last 3 years, have you been diagnosed or treated for: Other major mental illness |  |  |  | **✓** | **✓** | **✓** | **✓** |  |
| For the problems you had, did you seek help? Depression |  |  | **✓** | **✓** | **✓** | **✓** | **✓** | **✓** |
| For the problems you had, did you seek help? Episodes of intense anxiety (eg panic attacks) |  |  | **✓** | **✓** | **✓** | **✓** | **✓** | **✓** |
| For the problems you had, did you seek help? Other mental health problems |  |  |  | **✓** | **✓** | **✓** | **✓** | **✓** |
| Have you ever been told by a doctor that you have: Postnatal depression Yes, in the last 4 years |  | **✓** |  |  |  |  |  |  |
| Have you ever been told by a doctor that you have: Postnatal depression Yes, more than 4 years ago |  | **✓** |  |  |  |  |  |  |
| In the past three years, have you been diagnosed or treated for: Postnatal depression |  |  | **✓** | **✓** | **✓** | **✓** | **✓** | **✓** |
| **Cancer** |  |  |  |  |  |  |  |  |
| Have you ever been told by a doctor that you have: Cancer (Please specify type) | **✓** |  |  |  |  |  |  |  |
| Have you ever been told by a doctor that you have: Cancer (Please specify on page 29) Yes, in the last 4 years |  | **✓** |  |  |  |  |  |  |
| In the last 3 years, have you been diagnosed or treated for: Cancer |  |  | **✓** | **✓** |  |  |  |  |
| In the past three years, have you been diagnosed or treated for: Other cancer |  |  |  |  | **✓** | **✓** | **✓** | **✓** |
| Have you ever been diagnosed with or treated for: Breast cancer? |  |  |  |  |  |  |  | **✓** |
| Have you ever been diagnosed with or treated for cervical cancer? |  |  |  |  |  |  |  | **✓** |
| ***Other major illness** |  |  |  |  |  |  |  |  |
| Have you ever been told by a doctor that you have: Other major illness (Please specify on line) | **✓** |  |  |  |  |  |  |  |
| Have you ever been told by a doctor that you have: Other major illness (Please specify on page 29) Yes, in the last 4 years |  | **✓** |  |  |  |  |  |  |
| Have you ever been told by a doctor that you have: Other major illness (Please specify on page 29) Yes, more than 4 years ago |  | **✓** |  |  |  |  |  |  |
| In the past three years, have you been diagnosed or treated for: Other major illness |  |  | **✓** | **✓** | **✓** | **✓** | **✓** | **✓** |

* Note: ALSWH=Australian Longitudinal Study on Women’s Health. Other major illness and other major physical illness questions required participants to record their illness as free-text at the end of the survey.

**ALSWH 1989-95 cohort**

| **Survey question** | **S1** | **S2** | **S3** | **S4** | **S5** |
| --- | --- | --- | --- | --- | --- |
| **Diabetes** |  |  |  |  |  |
| Ever had insulin dependent (Type 1) diabetes | **✓** | **✓** |  |  |  |
| Ever had non-insulin dependent (Type 2) diabetes | **✓** | **✓** |  |  |  |
| Have you ever been diagnosed with or treated for: Type I Diabetes |  |  |  |  | **✓** |
| Have you been diagnosed with or treated for in the last 12 months? Type I Diabetes |  |  |  |  | **✓** |
| Have you ever been diagnosed with or treated for: Type II Diabetes |  |  |  |  | **✓** |
| Have you been diagnosed with or treated for in the last 12  months? Type II Diabetes |  |  |  |  | **✓** |
| **Cardiac disease** |  |  |  |  |  |
| Ever had hypertension (high blood pressure) | **✓** | **✓** |  |  |  |
| Ever had heart disease | **✓** | **✓** |  |  |  |
| **Asthma** |  |  |  |  |  |
| Ever had asthma | **✓** | **✓** | **✓** |  | **✓** |
| Diagnosed with or treated for asthma in the last 12 months |  |  |  |  | **✓** |
| In the last 12 months, have you had any of the following: Breathing difficulty | **✓** | **✓** | **✓** | **✓** | **✓** |
| **Thyroid disease** |  |  |  |  |  |
| Have you ever been diagnosed with or treated for: Thyroid condition |  |  |  |  | **✓** |
| Have you been diagnosed with or treated for in the last 12 months? Thyroid condition |  |  |  |  | **✓** |
| **Mental health conditions** |  |  |  |  |  |
| Ever had depression | **✓** | **✓** | **✓** |  | **✓** |
| Diagnosed with or treated for in the last 12 months? Depression |  |  |  |  | **✓** |
| Ever had an anxiety disorder | **✓** | **✓** | **✓** |  | **✓** |
| Diagnosed with or treated for in the last 12 months? Anxiety |  |  |  |  | **✓** |
| Ever had other mental illness | **✓** | **✓** | **✓** |  | **✓** |
| Diagnosed with or treated for in the last 12 months? Other major mental illness |  |  |  |  | **✓** |
| Ever been diagnosed or treated for: post-traumatic stress disorder (PTSD) |  | **✓** | **✓** |  | **✓** |
| Diagnosed with or treated for in the last 12 months? Post-traumatic stress disorder |  |  |  |  | **✓** |
| Ever been diagnosed or treated for: anorexia |  | **✓** | **✓** |  | **✓** |
| Diagnosed with or treated for in the last 12 months? anorexia |  |  |  |  | **✓** |
| Ever been diagnosed or treated for: bulimia |  | **✓** | **✓** |  | **✓** |
| Diagnosed with or treated for in the last 12 months? bulimia |  |  |  |  | **✓** |
| Ever been diagnosed or treated for: other eating disorder |  | **✓** | **✓** |  | **✓** |
| Diagnosed with or treated for in the last 12 months? Other eating disorder |  |  |  |  | **✓** |
| Ever been diagnosed or treated for: bipolar disorder |  | **✓** | **✓** |  | **✓** |
| Diagnosed with or treated for in the last 12 months? Bipolar disorder |  |  |  |  | **✓** |
| Ever been diagnosed or treated for: obsessive compulsive disorder |  | **✓** | **✓** |  |  |
| Ever been diagnosed or treated for: borderline personality disorder |  | **✓** | **✓** |  | **✓** |
| Diagnosed with or treated for in the last 12 months? borderline personality disorder |  |  |  |  | **✓** |
| **Cancer** |  |  |  |  |  |
| Ever had other cancer | **✓** |  |  |  |  |
| ***Other major illness** |  |  |  |  |  |
| Ever had major physical illness - Other major physical illness | **✓** | **✓** | **✓** |  |  |
| Ever had other (text) | **✓** |  |  |  |  |

* Note: ALSWH=Australian Longitudinal Study on Women’s Health. Other major physical illness questions required participants to record their illness as free-text at the end of the survey.
