# Supplementary material for: Increased chronic disease prevalence among the younger generation: Findings from a population-based data linkage study to inform chronic disease ascertainment among reproductive-aged Australian women
Source: PLoS One. 2021 Aug 18;16(8):e0254668. doi: 10.1371/journal.pone.0254668 (PMC8372972; doi:10.1371/journal.pone.0254668)
Supplement: S3 Table — (DOCX) [file pone.0254668.s003.docx]

**S3 Table. Case ascertainment algorithms**

**Case ascertainment algorithm for diabetes**

|  | **ELIGIBILITY CRITERIA** |
| --- | --- |
| **ALSWH SURVEYS** | Diabetes self-reported in at least two surveys |
| **MBS** | Reported once or more for diabetes annual cycle; eye exam, specific for patients with established diabetes; and allied health group service, specific for patients with established diabetes  **OR**  *2 or more HbA1c tests related to management of established diabetes within a 12- month period |
| **PBS**^🟄^ | Reported twice or more within a 12-month period in two calendar years |
| **COD** | Reported once |
| **APDC** | Reported once or more |

Note: ALSWH=Australian Longitudinal Study on Women’s Health; MBS=Medicare Benefits Schedule; PBS=Pharmaceuticals Benefits Scheme; COD=Cause of Death (from the National Death Index); APDC=Admitted Patient Data Collection.

* The MBS Service Incentive Program for diabetes care requires at least one HbA1c measurement per year. It is recommended that HbA1c testing is done every 6 months if meeting targets, or every 3 months if targets are not being met or if therapy has changed. Therefore, at least 2 HbA1c tests within a 12-month period was required for inclusion.

^ç^PBS: Excluding times of pregnancy (defined as 40 weeks), based on child’s dates of birth. This was to exclude women who may have been diagnosed & treated for gestational diabetes.

**Case ascertainment algorithm for cardiac disease**

|  | **ELIGIBILITY CRITERIA** |
| --- | --- |
| **ALSWH SURVEYS** | Cardiac disease self-reported in at least two surveys |
| **MBS** | Reported once or more |
| **PBS**^🟄^ | Reported twice or more within a 12-month period in two calendar years |
| **COD** | Reported once |
| **APDC** | Reported once or more |

Note: ALSWH=Australian Longitudinal Study on Women’s Health; MBS=Medicare Benefits Schedule; PBS=Pharmaceuticals Benefits Scheme; COD=Cause of Death (from the National Death Index); APDC=Admitted Patient Data Collection.

^🟄^PBS: Excluding times of pregnancy (based on child’s dates of birth). Based on SOMANZ guidelines regarding first- and second-line treatment for hypertension in pregnancy, the following medications were excluded from 20-weeks: Methyldopa (C02AB01; C02AB02); Clonidine (C02AC01); Labetalol (C07AG01); Oxprenolol (C07AA02); Hydralazine (C02DB02); Nifedipine (C08CA05); Prazosin (C02CA01)

**Case ascertainment algorithm for chronic kidney disease**

|  | **ELIGIBILITY CRITERIA** |
| --- | --- |
| **ALSWH SURVEYS** | Chronic kidney disease self-reported in at least two surveys |
| **MBS** | Reported once or more |
| **PBS** | Reported twice or more within a 12-month period in two calendar years |
| **COD** | Reported once |
| **APDC** | Reported once or more |

Note: ALSWH=Australian Longitudinal Study on Women’s Health; MBS=Medicare Benefits Schedule; PBS=Pharmaceuticals Benefits Scheme; COD=Cause of Death (from the National Death Index); APDC=Admitted Patient Data Collection.

**Case ascertainment algorithm for asthma**

|  | **ELIGIBILITY CRITERIA** |
| --- | --- |
| **ALSWH SURVEYS** | 1973-78 cohort: Asthma self-reported in at least two surveys (excluding survey 1)  1989-95 cohort: Asthma self-reported in at least two surveys and reported breathing difficulties sometimes/often at the same surveys |
| **MBS** | Reported once or more |
| **PBS** | Reported twice or more within a 12-month period in two calendar years |
| **COD** | Reported once |
| **APDC** | Reported once or more |

Note: ALSWH=Australian Longitudinal Study on Women’s Health; MBS=Medicare Benefits Schedule; PBS=Pharmaceuticals Benefits Scheme; COD=Cause of Death (from the National Death Index); APDC=Admitted Patient Data Collection.

**Case ascertainment algorithm for autoinflammatory arthropathies and connective tissue disease**

|  | **ELIGIBILITY CRITERIA** |
| --- | --- |
| **ALSWH SURVEYS** | Autoinflammatory arthropathies and connective tissue disease self-reported in at least two surveys |
| **PBS** | Reported twice or more within a 12-month period in two calendar years |
| **COD** | Reported once |
| **APDC** | Reported once or more |

Note: ALSWH=Australian Longitudinal Study on Women’s Health; MBS=Medicare Benefits Schedule; PBS=Pharmaceuticals Benefits Scheme; COD=Cause of Death (from the National Death Index); APDC=Admitted Patient Data Collection.

**Case ascertainment algorithm for inflammatory bowel disease**

|  | **ELIGIBILITY CRITERIA** |
| --- | --- |
| **ALSWH SURVEYS** | Inflammatory bowel disease self-reported in at least two surveys |
| **MBS** | One or more relevant MBS items |
| **PBS** | Reported twice or more within a 12-month period in two calendar years |
| **COD** | Reported once |
| **APDC** | Reported once or more |

Note: ALSWH=Australian Longitudinal Study on Women’s Health; MBS=Medicare Benefits Schedule; PBS=Pharmaceuticals Benefits Scheme; COD=Cause of Death (from the National Death Index); APDC=Admitted Patient Data Collection.

**Case ascertainment algorithm for thyroid disease**

|  | **ELIGIBILITY CRITERIA** |
| --- | --- |
| **ALSWH SURVEYS** | Thyroid disease self-reported in at least two surveys |
| **PBS** | Reported twice or more within a 12-month period in two calendar years |
| **COD** | Reported once |
| **APDC** | Reported once or more |

Note: ALSWH=Australian Longitudinal Study on Women’s Health; MBS=Medicare Benefits Schedule; PBS=Pharmaceuticals Benefits Scheme; COD=Cause of Death (from the National Death Index); APDC=Admitted Patient Data Collection.

**Case ascertainment algorithm for multiple sclerosis**

|  | **ELIGIBILITY CRITERIA** |
| --- | --- |
| **ALSWH SURVEYS** | Multiple sclerosis self-reported in at least two surveys |
| **PBS** | Reported twice or more within a 12-month period in two calendar years |
| **COD** | Reported once |
| **APDC** | Reported once or more |

Note: ALSWH=Australian Longitudinal Study on Women’s Health; MBS=Medicare Benefits Schedule; PBS=Pharmaceuticals Benefits Scheme; COD=Cause of Death (from the National Death Index); APDC=Admitted Patient Data Collection.

**Case ascertainment algorithm for mental health conditions**

|  | **ELIGIBILITY CRITERIA** |
| --- | --- |
| **ALSWH SURVEYS** | Mental health condition self-reported in at least two surveys |
| **MBS** | One or more relevant MBS items |
| **PBS**^🟄^ | Reported twice or more within a 12-month period in two calendar years |
| **COD** | Reported once |
| **APDC** | Reported once or more |

Note: ALSWH=Australian Longitudinal Study on Women’s Health; MBS=Medicare Benefits Schedule; PBS=Pharmaceuticals Benefits Scheme; COD=Cause of Death (from the National Death Index); APDC=Admitted Patient Data Collection.

^🟄^PBS: Excluding times of pregnancy (defined as 40 weeks), based on child’s dates of birth. This should exclude women who were diagnosed with antenatal depression and anxiety).

**Case ascertainment algorithm for cancer**

|  | **ELIGIBILITY CRITERIA** |
| --- | --- |
| **Cancer registry** | Reported once or more |
| **ALSWH SURVEYS** | Cancer (excluding non-melanotic skin) self-reported in at least two surveys |
| **COD** | Reported once |
| **APDC** | Reported once or more |

Note: ALSWH=Australian Longitudinal Study on Women’s Health; COD=Cause of Death (from the National Death Index); APDC=Admitted Patient Data Collection.
