# Supplementary material for: Increased chronic disease prevalence among the younger generation: Findings from a population-based data linkage study to inform chronic disease ascertainment among reproductive-aged Australian women
Source: PLoS One. 2021 Aug 18;16(8):e0254668. doi: 10.1371/journal.pone.0254668 (PMC8372972; doi:10.1371/journal.pone.0254668)
Supplement: S4 Table — (DOCX) [file pone.0254668.s004.docx]

**S4 Table. Baseline characteristics for eligible women (aged 18-23 years) from two cohorts of the Australian Longitudinal Study on Women’s Health^a^**

| **Characteristic** | **Born 1973-78**  **Baseline: 1996**  **N=13,501** | | **Born 1989-95**  **Baseline: 2012/13**  **N=16,964** | |
| --- | --- | --- | --- | --- |
|  | **N** | **%** | **N** | **%** |
| Country of birth |  |  |  |  |
| Australia | 11,650 | 86.7 | 15,318 | 92.0 |
| Other English-speaking background | 693 | 5.1 | 559 | 3.4 |
| Non-English-speaking background | 1097 | 8.2 | 768 | 4.6 |
| *Missing* | *61* |  | *319* |  |
| Relationship status |  |  |  |  |
| Partnered | 3046 | 22.7 | 4719 | 28.1 |
| Unpartnered | 10,390 | 77.3 | 12,062 | 71.9 |
| *Missing* | *65* |  | *183* |  |
| Area of residence |  |  |  |  |
| Major cities | 7000 | 51.9 | 12751 | 75.2 |
| Inner regional | 4070 | 30.2 | 2890 | 17.0 |
| Outer regional/remote/very remote | 2427 | 18.0 | 1315 | 7.8 |
| *Missing* | *4* |  | *8* |  |
| Educational attainment |  |  |  |  |
| Year 12 or below | 9529 | 71.0 | 8557 | 51.0 |
| Certificate/diploma | 2401 | 17.9 | 4556 | 27.1 |
| Tertiary/post-graduate | 1494 | 11.1 | 3668 | 21.9 |
| *Missing* | *77* |  | *183* |  |
| Income management |  |  |  |  |
| Impossible/difficult all the time | 2498 | 18.6 | 4254 | 25.4 |
| Difficult some of the time | 4464 | 33.2 | 5978 | 35.6 |
| Not too bad/easy | 6490 | 48.2 | 6544 | 39.0 |
| *Missing* | *49* |  | *188* |  |
| Self-rated health |  |  |  |  |
| Excellent | 1667 | 12.4 | 1088 | 6.5 |
| Very good/good | 10119 | 75.4 | 12876 | 76.6 |
| Fair/poor | 1637 | 12.2 | 2836 | 16.9 |
| *Missing* | *78* |  | *164* |  |
| Body mass index |  |  |  |  |
| Underweight | 1667 | 9.8 | 1288 | 7.9 |
| Healthy weight | 10119 | 68.3 | 9736 | 59.5 |
| Overweight | 1637 | 15.4 | 3147 | 19.2 |
| Obese | 761 | 6.5 | 2183 | 13.3 |
| *Missing* | *1740* |  | *610* |  |
| Alcohol consumption |  |  |  |  |
| Low risk drinker | 6844 | 51.3 | 9319 | 55.5 |
| Non-drinker | 1187 | 8.9 | 1261 | 7.5 |
| Rarely drinks | 4591 | 34.4 | 5596 | 33.3 |
| Risky/high-risk drinker | 730 | 5.5 | 622 | 3.7 |
| *Missing* | *149* |  | *166* |  |
| Smoking status |  |  |  |  |
| Non-smoker | 6727 | 52.1 | 13635 | 81.2 |
| ^b^Ex-smoker | 1976 | 15.3 | 0 | 0.0 |
| Current smoker | 4217 | 32.6 | 3163 | 18.8 |
| *Missing* | *581* |  | *166* |  |

^a^ Eligible and provided consent for data linkage with administrative health datasets

^b^ Smoking status for the 1989-95 cohort was based on a question which asked about current smoking with the options being daily, less than daily and not at all.

*Some percentages do not equal 100 due to rounding.
